# Oocyte Scoring Enhances Embryo-Scoring in Predicting Pregnancy Chances with IVF Where It Counts Most

**DOI:** 10.1371/journal.pone.0143632

**Published:** 2015-12-02

**Authors:** Emanuela Lazzaroni-Tealdi, David H. Barad, David F. Albertini, Yao Yu, Vitaly A. Kushnir, Helena Russell, Yan-Guang Wu, Norbert Gleicher

**Affiliations:** 1 The Center for Human Reproduction, New York, New York, United States of America; 2 The Foundation for Reproductive Medicine, New York, New York, United States of America; 3 Department of Obstetrics and Gynecology, Albert Einstein College of Medicine, Bronx, New York, United States of America; 4 Department of Molecular and Integrative Physiology, University of Kansas Medical Center, Kansas City, Kansas, United States of America; 5 Department of Obstetrics and Gynecology, Wake Forest University, Winston Salem, North Carolina, United States of America; 6 School of Health Professions, Eastern Virginia Medical School, Norfolk, Virginia, United States of America; 7 Stem Cell and Molecular Embryology Laboratory, The Rockefeller University, New York, New York, United States of America; University of Kansas Medical Center, UNITED STATES

## Abstract

**Context:**

Our center’s quality improvement optimization process on many occasions anecdotally suggested that oocyte assessments might enhance embryo assessment in predicting pregnancy chances with in vitro fertilization (IVF).

**Objective:**

To prospectively compare a morphologic oocyte grading system to standard day-3 morphologic embryo assessment.

**Design, Setting, Patients:**

We prospectively investigated in a private academically-affiliated infertility center 94 consecutive IVF cycles based on 6 criteria for oocyte quality: morphology, cytoplasm, perivitelline space (PVS), zona pellucida (ZP), polar body (PB) and oocyte size, each assigned a value of -1 (worst), 0 (average) or +1 (best), so establishing an average total oocyte score (TOS). Embryo assessment utilized grade and cell numbers of each embryo on day-3 after oocyte retrieval. Clinical pregnancy was defined by presence of at least one intrauterine gestational sac.

**Interventions:**

Standard IVF cycles in infertile women.

**Main Outcome Measures:**

Predictability of pregnancy based on oocyte and embryo-grading systems.

**Results:**

Average age for all patients was 36.5 ± 7.3 years; mean oocyte yield was 7.97± 5.76; Patient specific total oocyte score (PTOS) was -1.05 ± 2.24. PTOS, adjusted for patient age, was directly related to odds of increased embryo cell numbers (OR 1.12, P = 0.025), embryo grade (OR 1.19, P < 0.001) and clinical pregnancy [OR 1.58 (95%CI 1.23 to 2.02), P < 0.001]. Restricting the analysis to day three embryos of high quality (8-cell/ good grades), TOS was still predictive of clinical pregnancy (OR 2.08 (95%CI 1.26 to 3.44, P = 0.004). Among the 69 patients with embryos of Grade 4 or better available for transfer 23 achieved Clinical Pregnancy. When the analysis was restricted to the 69 transfers with good quality embryos (≥ Grade 4) the Oocyte Scoring System (TOS) (AUC±SE 0.863±0.044, oocyte score) provided significantly greater predictive value for clinical pregnancy compared to the embryo grade alone (AUC 0.646 ± 0.072, embryo grade) p = 0.015.

**Conclusions:**

Oocyte-scoring, thus, provides useful clinical information especially in good prognosis patients with large numbers of high quality embryos. This finding appears of particular importance at a time when many IVF centers are committing sizable investments to closed incubation systems with time-lapse photography, which are exclusively meant to define embryo morphology.

## Background

The relationship between oocyte quality, embryo development, and in vitro fertilization (IVF) outcomes has been widely studied [1–10]. Most IVF programs, ours included, rely almost exclusively on embryo morphology in selecting embryos for transfer.

In our center’s weekly review of failed IVF cycles we found that oocyte assessments on many occasions predicted IVF outcomes better than embryo assessments. We hypothesized that determining pregnancy potential at oocyte rather than embryo level might provide further insight in embryo selection for transfer. The present prospective study was designed to test this hypothesis.

Because embryo quality is currently widely considered the best available prediction model for pregnancy chances, research efforts have almost exclusively concentrated on improving embryo rather than oocyte assessments. Recently, this has led to increasing utilization of costly closed automated incubation systems, involving time-lapse photography [11].

Considering already very high IVF costs, economics would warrant more cost-effective embryo selection. We, therefore, in this study prospectively investigated a simple, novel oocyte scoring system, and compared it to traditional day 3 embryo assessments as still practiced by most IVF laboratories.

## Materials and Methods

### Patient population

We prospectively investigated a cohort of 94 patients undergoing IVF cycles between Nov. 1, 2011 and Jan. 31, 2013. Fourteen donor egg cycles were eligible and included in this analysis. In the case of donor cycles the egg donor’s age was used in the analysis.

Patients of all races and ages were included in the study. Women with less than 7.0 mm endometrial thickness on day of embryo transfer were excluded. Only intracytoplasmic sperm insertion (ICSI) cycles and patients undergoing embryo transfer (ET) on day-3 after fertilization were considered. Patients with less than 2 mature oocytes were excluded.

Other exclusion criteria were PGD (preimplantation genetic diagnosis) cycles, elective complete cryopreservation, day-5 ETs, cycles from cryopreserved oocytes.

### Study Design

This study was designed as a prospective cohort study to determine the association of oocyte scores with traditional embryo scoring and to test whether oocyte scoring, in addition to embryo grade, could further contribute to prediction of IVF cycle outcome. Thus, all MII oocytes were scored as described below, and for those resulting in day three embryos, relationships between the two systems and associations with IVF outcomes were assessed.

### Oocyte preparation and assessment

Oocytes were routinely incubated for at least 2 hours in an organ dish, containing Human Tubal Fluid (HTF) media (LifeGlobal, LLC, Gilford, CT) with 10% of Human Serum Albumin (HSA, LifeGlobal, LLC, Gilford, CT) after retrieval. They then were stripped of cumulus cells, using 80 IU/ml hyaluronydase (LifeGlobal, LLC, Gilford, CT), and were carefully mechanically denuded. Intracytoplasmic sperm injection (ICSI) was only performed on mature oocytes. For the ICSI procedure oocytes were placed into an ICSI dish containing 25ul drops of HTF with HEPES, with 10% of HSA added under mineral oil (LifeGlobal, LLC, Gilford, CT). Each drop was labeled with a number. Following the ICSI procedure, each oocyte was cultured individually under mineral oil in labeled drops (100ul) of Blastocyst media (LifeGlobal, LLC, Gilford, CT) with 10% HSA. Each dish was prepared the day before oocyte retrieval.

All available mature oocytes were assessed for quality before performing ICSI by digital imaging of the oocyte just before sperm injection (INFINITY software, Lumenera Corp, Ottawa, Ontario, Canada). Individual oocytes were placed into a single-numbered drop in an ICSI dish, allowing for longitudinal assessments of subsequent fertilization and embryo development. A stage micrometer, connected to an ICSI microscope, measured oocyte diameter. Total oocyte scores (TOS) were then stored in the laboratory’s database, protected by a password chosen by the laboratory director. These scores were not modified after fertilization since the score is designed to represent the pre-fertilized oocyte.

### Total Oocyte Score (TOS)

Individual oocytes were evaluated based on 6 parameters: (i) Oocyte shape [12]; (ii) oocyte size [13]; (iii) ooplasm characteristics [14–20]; (iv) structure of the perivitelline space (PVS) [21,22]; (v) zona pellucida (ZP) [23,24]; and (vi) polar body (PB) morphology [25–27]. Each parameter was graded as worst (-1), average (0), or best (1), creating a TOS by adding up individual parameter assessments. The maximal TOS of an oocyte, therefore, could be a +6, the lowest a -6.

**Fig 1:**
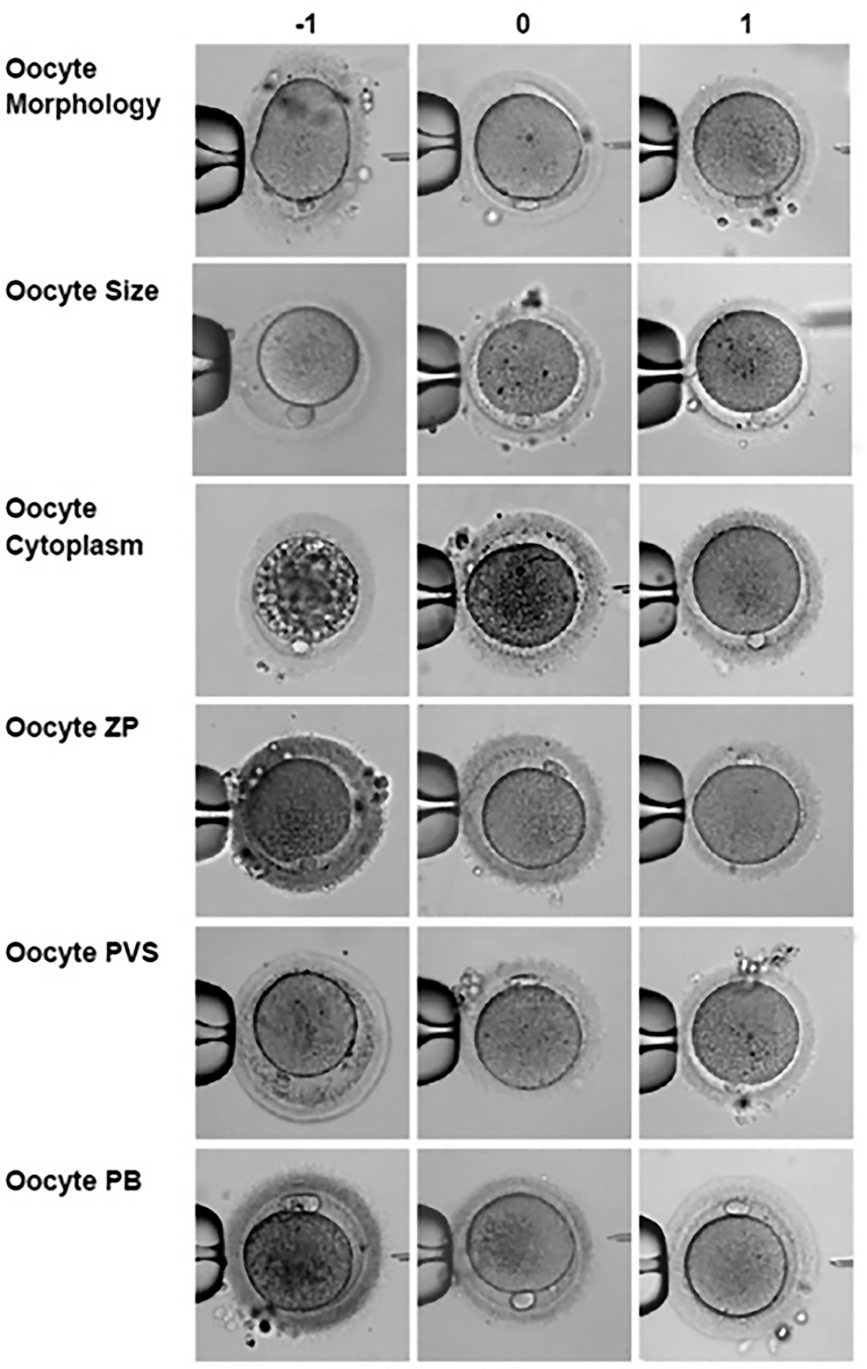
Example photographs for oocyte scoring system. The figure represents the oocyte grading system for the six morphological characteristics analyzed in this project (Morphology, Size, Cytoplasm, ZP, PVS and PB). For each oocyte, each single characteristic was graded as worst (-1), average (0), or best (1) creating a TOS by adding up individual parameter assessments.

The 6 individual parameters were assessed as follows (Fig 1): (i) If oocyte morphology was poor (dark general oocyte coloration and/or ovoid shape), it was assigned a value of -1; if almost normal (less dark general oocyte coloration and less ovoid shape), it was assigned a value of 0; if it was judged to be normal, it was assigned a value of + 1.

(ii) If oocyte size was defined as abnormally small or large, it was assigned a value -1 if size was below 120μ or greater 160μ. If the size was almost normal, i.e., did not deviate from normal by more than 10 μ, a value of 0 was assigned, and a value of + 1 was assigned if oocyte size was within normal range > 130 μ and <150 μ.

(iii) If the ooplasm was very granular and/or very vacuolated and/or demonstrated several inclusions, a value of -1 was assigned. If it was only slightly granular and/or demonstrated only few inclusions, a value of 0 was assigned. Absence of granularity and inclusions resulted in a +1 value.

(iv) The PVS was defined as -1 with an abnormally large PVS, an absent PVS or a very granular PVS. It was assigned a value of 0 with a moderately enlarged PVS and/or small PVS and/or a less granular PVS. A value of +1 was assigned to a normal size PVS with no granules.

(v) If ZPs were very thin or thick (<10μ or >20μ) the oocyte was assigned a -1. If the ZP did not deviate from normal by more than 2 μ it was assigned 0. A normal zona (> 12 μ and <18 μ) was assigned a +1.

Finally, (vi) PB morphology was defined as follows: Flat and/or multiple PBs, granular and/or either abnormally small or large PBs were designated as -1. PBs, judged a fair but not excellent were designated as 0, and a designation of +1 was given to PBs of normal size and shape.

### Fertilization assessment

A first fertilization check was performed at approximately 18 hours after the intracytoplasmic sperm injection (ICSI) procedure on day-1 post-fertilization by checking under the microscope for presence of two pronuclei (PN). Following the numerical order in the culture dish, pictures of and notes on each oocyte were taken. The culture dish containing the zygotes was then incubated uninterrupted until day-3, the day of the embryo transfer.

### Embryo assessment

Each embryo was individually evaluated under the microscope early in the morning of day-3. We elected to use Day 3 embryo morphology for comparison because our patients were undergoing a day 3 transfer. Embryo assessments were performed by counting blastomeres, assessing embryo shape, and determining the degree of fragmentation. Embryos were graded based on the following parameters: Excellent or Grade 5, reached at least 8 cells with <5% fragmentation; Good or Grade 4, reached 6–8 cells with ≤5% fragmentation; Average or Grade 3, 6–8 cells with 5–20% fragmentation; Poor or Grade 2, reached 4–8 cells with 20–40% fragmentation; Very poor or Grade 1, 4–8 cells, with >40% fragmentation. For purpose of analysis Embryo Grade was treated as an ordinal variable with 5 levels and Cell number as a continuous variable reflected the cell count on day three.

Embryos selected for transfer were incubated in the transfer dish until they were removed from the incubator for loading into the transfer catheter. Patients maintained bed rest for approximately 20 minutes after embryo transfer.

### IVF cycle outcome assessment

Each IVF cycle was entered into an anonymized electronic research database, which contained detailed patient characteristics and laboratory data.

Clinical pregnancy was defined as the presence of at least one gestational sac in the uterus. Implantation rates were defined based on visualized gestational sacs on ultrasound examinations per embryo transferred. Associations of implantation and clinical pregnancy rates with individual and cumulative oocyte, as well as embryo characteristics were individually and cumulatively assessed.

### Statistical analysis

We first assessed the association of oocyte morphology with embryo morphology. Next, we assessed the association of oocyte and embryo quality with chance of implantation and clinical pregnancy by assessing individual oocytes and embryos, using quantitative and qualitative techniques adjusted for BMI, race, and age.

Differences between continuous and categorical data were compared using Kruskal-Wallis, Mann-Whitney U, and Chi-Square tests. To account for the dependence among eggs collected from the same individual, we used Generalized Estimating Equations (GEE) models.

Multivariate linear regression models were employed to study effects of average quality of eggs on average quality of embryos transferred. We also explored the relationship between average quality of eggs and occurrence of clinical pregnancy, using multinomial logistic regression.

Implantation rates were compared among three categories of oocyte scores using General Linear Model adjusted for age.

ROC curves were built to compare the predictive value of the oocyte and embryo scoring systems for clinical IVF pregnancy. ROC curves were constructed with and without weighting based on numbers of embryos transferred. Finally, ROC curves were constructed based on whether good (≥8 cells) or poor quality (<8 cells) embryos were transferred.

All Analyses were adjusted for age. Using SAS 9.2, all analyses were conducted by the center’s senior statistician (Y.Y) and by D.H.B. Findings were considered statistically significant at P< 0.05

### Institutional Review Board

All patients at our center at the time of initial presentation sign an informed consent that allows for use of their medical record data for research purposes, as long as those data remain confidential and the patient’s anonymity is maintained. Use of the center’s electronic research data bank guarantees both. Studies making use of this bank are, therefore, exempt from routine Institutional Review Board (IRB) review, and require only expedited review, which was obtained for this study.

Electronic records of patients selected for this study were already anonymized, and were assigned random study numbers. Study data were transferred to a Microsoft Excel spreadsheet and statistically analyzed in collaboration with the Section of Medical Statistics at the center.

## Results

This study involved 594 mature oocytes from 94 patients undergoing IVF. Average age of patients was 39.8 ± 5.6 years. (Supplemental data for each oocyte and each patient is available in S1 Table and S2 Table.) Patient characteristics are summarized in Table 1. The number of eggs collected per retrieval was 7.97± 5.76. The average number of embryos transferred to each patient was 2.53 ± 1.06 (range 1 to 7). The median embryo grade was 4.33 (range 3 to 5). Of the 594 mature oocytes, 383 developed into embryos with 8 or more cells on day 3 (343 eight cell; 29 ten-cell; 11 twelve-cell) of which 318 were grade 4 or 5. Thus, 318 good quality embryos and 276 poorer quality embryos were in the analysis.

**Table 1:** Patient characteristics.

	n	Mean or %	Std. Deviation
*Age (years)*	94	36.6	7.3
*Height (inches)*	94	64.4	3.0
*Weight (pounds)*	94	146.9	37.1
*Gravida*	94	1.53	1.99
*Para*	94	0.30	0.78
*SAB*	94	0.97	1.41
*FSH (mIU/ml)*	94	13.1	12.5
*AMH (ng/mL)*	94	1.39	1.90
*Total FSH dose (IU)*	94	5941	2698
*Embryos transferred*	94	2.53	1.07
*Diagnosis* *			
*Male Factor*	7	7.6%	
*Endometriosis*	3	3.3%	
*PCO*	5	5.4%	
*DOR*	75	79.3%	
*Tubal factor*	16	17.4%	
*Uterine factor*	7	7.6%	
*Other*	22	23.9%	
*Unexplained*	1	1.1%	
*Race*			
*Asian*	17	18.1%	
*Black*	16	17%	
*White*	61	64.9%	

* Patients may have more than one diagnosis

**Fig 2:**
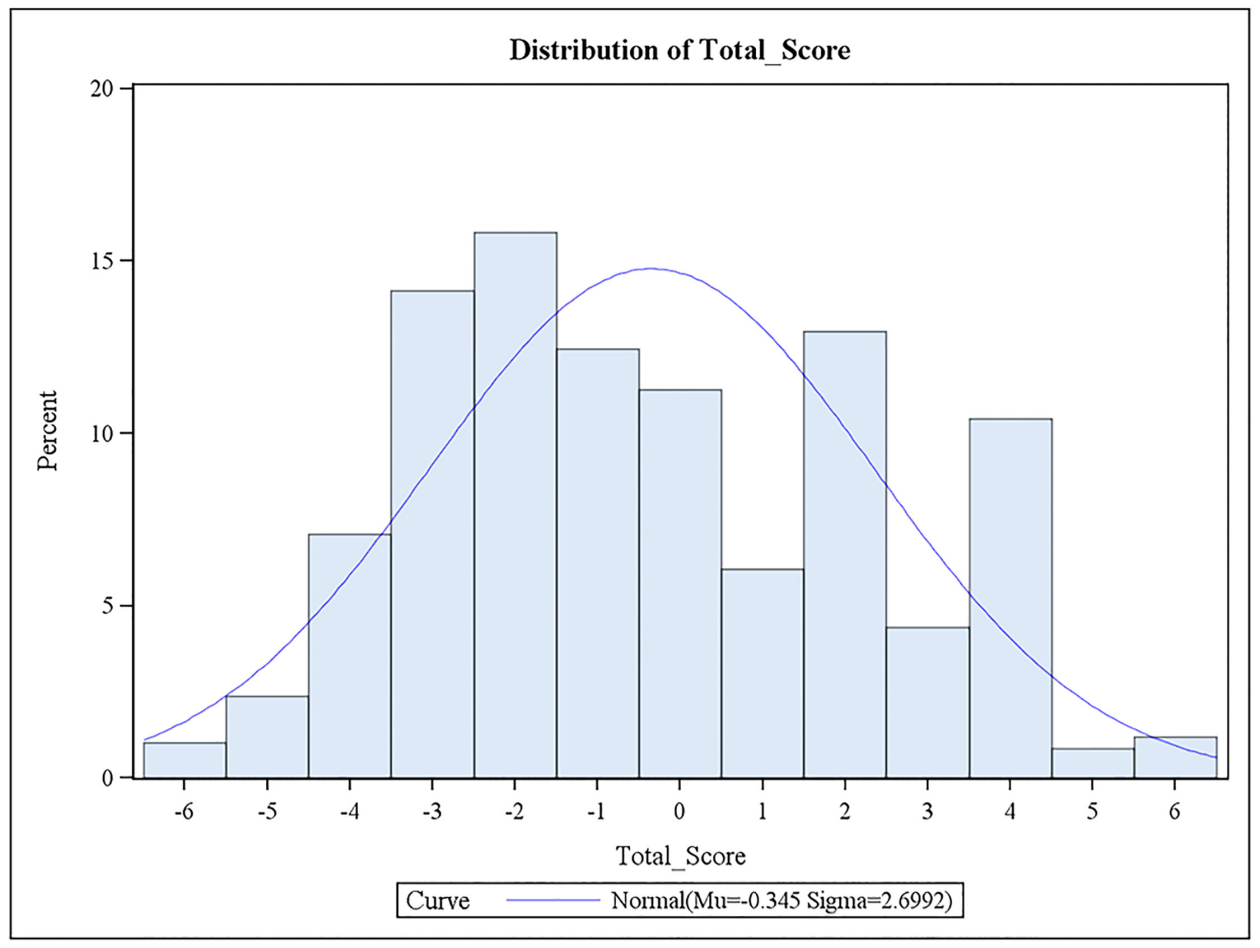
Distribution of TOS among studied patients. Fig 2 is the histogram of Total Score. The Total Score distribution is not symmetric.

Oocyte characteristics of all patients are shown in Table 2. The six parameters considered in the model to evaluate oocyte quality (morphology, cytoplasm, PVS, ZP, PB and oocyte size) were each differently distributed (Table 2). TOSs were distributed with a moderate skew to the left (Fig 2). The average TOS was -0.345 ± 2.67.

**Table 2:** Characteristics of 594 oocytes.

Oocyte Characteristics*	1	0	-1
Overall morphology	210(35.4%)	243(40.9%)	141(23.7%)
Size of oocyte	247(41.6%)	216(36.4%)	131(22.1%)
Patterns of cytoplasm	35(5.9%)	156(26.3%)	403(67.9%)
Size of PVS and presence of granules	85(14.3%)	123(20.7%)	386(65.0%)
Thickness of zona pellucida	316(53.2%)	191(32.2%)	87(14.7%)
Morphology of polar body	228(38.5%)	187(31.5%)	179(30.1%)

* Graded levels of oocyte quality with +1 being best, 0 next best and -1 worst

Individual TOSs from all mature oocytes, were averaged for each patient to create a patient-specific TOS (PTOS). The average of all PTOS was -1.05 ± 2.24.

Table 3 demonstrates the relationship between oocyte quality and embryo development by summarizing results of univariate GEE models, assessing the effects of each of the six judged oocyte characteristics on resulting day-3 embryo cell numbers. The table shows raw odds ratios (OR) and OR adjusted for patient ages.

**Table 3:** Univariate associations of oocyte characteristics with Day 3 embryo ≥ 8 cells.

Variables	Level*	Unadjusted		Adjusted for Age	
		OR	P	OR	P
Overall morphology	0	1.29	0.42	1.16	0.63
	1	2.45	0.004	1.88	0.07
Size of oocyte	0	2.02	0.02	1.92	0.02
	1	2.48	0.003	2.11	0.02
Cytoplasm	0	1.52	0.14	1.44	0.19
	1	2.82	0.04	2.07	0.19
Size of PVS and presence of granules	0	1.07	0.83	1.04	0.88
	1	1.32	0.36	1.11	0.74
Thickness of zona pellucida	0	1.31	0.35	1.29	0.4
	1	1.88	0.06	1.42	0.34
Polar body morphology	0	1.6	0.11	1.45	0.19
	1	1.69	0.02	1.51	0.06

* Graded levels of quality with +1 being best, 0 next best and -1 worst; All associations are referenced to -1

As these data demonstrate, oocytes with better morphology, size, cytoplasm patterns and polar body morphology were more likely to result in day-3 embryos of good quality, i.e., ≥ 8 cells. Not surprisingly, increasing age is in all of these models also associated with decreased day 3-cell numbers of embryos. After adjustment for age, oocyte size was the only factor independently remaining associated with improved day-3 embryo cell numbers, although oocyte morphology and PB score approached significance.

The cumulative effect of these 6 oocyte characteristics was investigated by fitting a GEE model between day-3 embryo cell numbers and TOSs. Doing so, TOS was significantly associated with higher day-3 embryo cell numbers (OR 1.12, P = 0.025). In other words, for every 1-point increase in TOS, the odds of having an embryo with ≥ 8 cells increased by 12%. In a multivariate GEE model, with all oocyte characteristics adjusted for patient age, only oocyte size (P = 0.02), and of course age (P = 0.03), were significant predictors of good quality day-3 embryos with cell number ≥ 8.

This suggests that the predictive value of oocyte size for day 3-embryo cell numbers is independent of any age effect, while the other five oocyte characteristics are influenced by patient age.

Table 4 demonstrates that oocytes with best scores (level “1”), morphology, size, cytoplasm patterns and PB morphology are all individually significantly associated with improved day-3 embryo grades. For oocyte morphology, the odds ratio estimate is 2.87, indicating that an oocyte with normal morphology has 2.87 times higher odds of obtaining a higher grade day-3 embryo than oocytes with poor morphology (dark cytoplasm and/or ovoid shape).

**Table 4:** Univariate associations of oocyte characteristics with Day 3 embryo Grade.

Variables	Level*	Unadjusted		Adjusted for Age	
		OR	P	OR	P
Overall morphology	0	1.28	0.3	1.25	0.37
	1	2.87	< .0001	2.73	< .0001
Size of oocyte	0	1.55	0.15	1.51	0.17
	1	2.73	< .0001	2.61	< .0001
Cytoplasm	0	1.58	0.09	1.55	0.11
	1	3.23	< .0001	2.94	0.002
Size of PVS and presence of granules	0	0.85	0.56	0.83	0.51
	1	1.27	0.47	1.19	0.62
Thickness of Zona Pellucida	0	1.3	0.36	1.3	0.38
	1	2.44	< .0001	2.34	0.002
Polar Body Morphology	0	1.23	0.45	1.17	0.55
	1	1.75	0.01	1.69	0.02

* Graded levels of quality with +1 being best, 0 next best and -1 worst; All associations are referenced to -1

Results of size, cytoplasm, ZP and PB assessments can be interpreted similarly according to odds listed in Table 4. Interestingly, normal oocyte morphology, size, cytoplasm patterns and PB morphology remain highly significantly associated with good embryo grade even after adjustment for age (Table 4). In a multivariate GEE model, with all oocyte characteristics adjusted for age only oocyte size (P = 0.02), zona pellucida thickness (P = 0.03) were significant independent predictors of high grade day-3 embryos.

The cumulative effect of these 6 oocyte characteristics was investigated by fitting a GEE model between day-3 embryo grade and TOS. TOS was significantly associated with higher day-3 embryo grade (OR 1.19, P < 0.0001).

Among 94 IVF cycles studied, 29 (31%) resulted in ongoing clinical pregnancy, characterized by at least one gestational sac in uterus. Logistic regression adjusted for age demonstrated a significant relationship between the average PTOS and clinical pregnancy (OR 1.58 (95%CI 1.23 to 2.03); P < 0.001).

In an analysis of cycles in which only 8-cell embryos were transferred, logistic regression, adjusted for patient age, demonstrated that the odds ratio of clinical pregnancy among cycles having 8-cell embryos transferred was significantly increased with higher average PTOS (OR 2.08 (95%CI 1.26 to 3.44; P = 0.004).

Linear regression adjusted for age also found that average PTOS was significantly associated with higher average grade (higher cell count and less fragmentation) embryos available for transfer (Z = 3.43; P = 0.001).

PTOS was divided into three equal categories. Implantation rates in the three categories were Low PTOS 4.7%, Medium PTOS 7.1% and Best PTOS 44.8%. The Best PTOS category was significantly better that that in the two poorer categories (p < 0.001).

**Fig 3:**
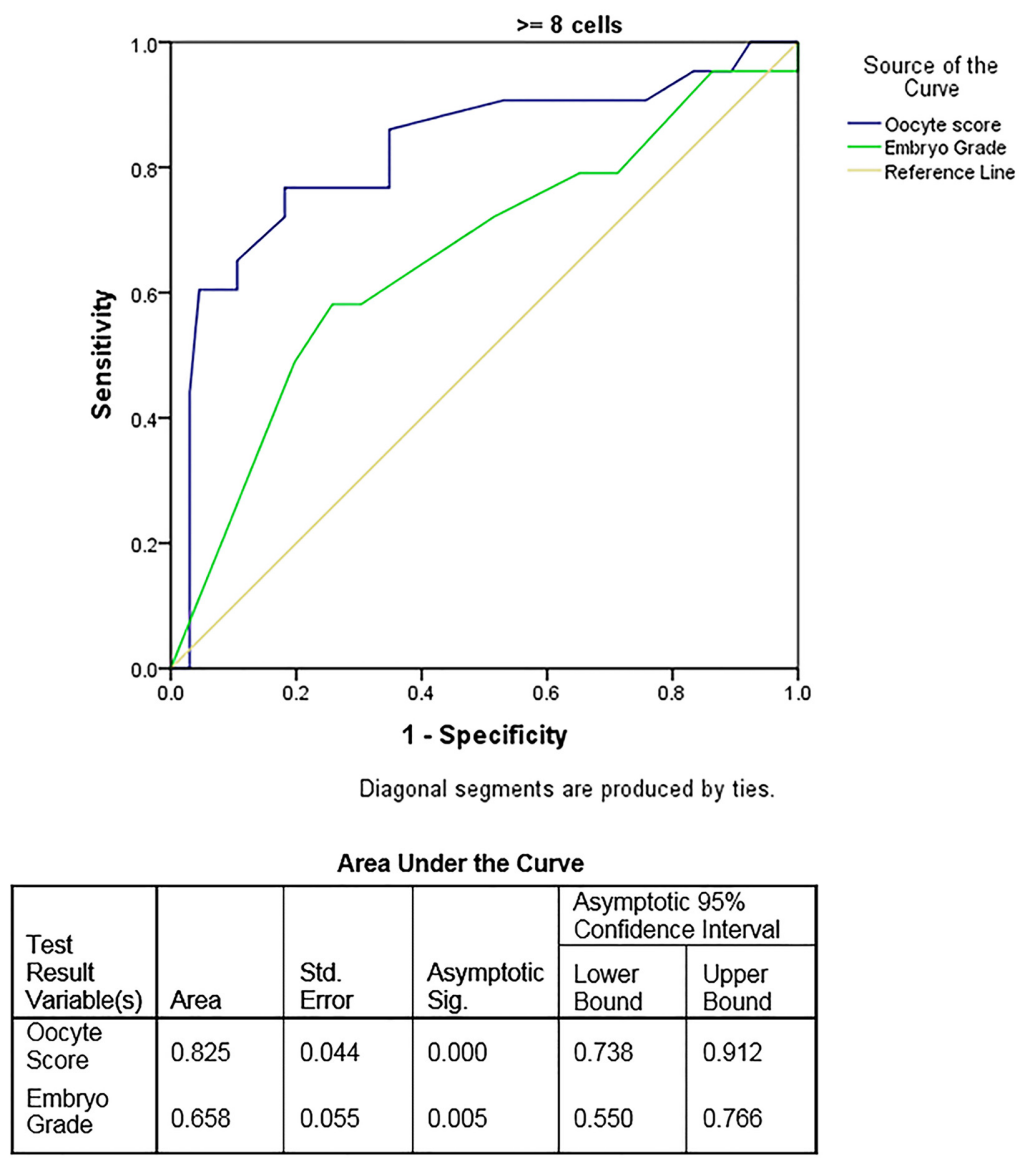
ROC curves for oocyte and embryo scores. Among the 69 patients with embryos of Grade 4 or better available for transfer 23 achieved Clinical Pregnancy. When the analysis was restricted to the 69 transfers with Good quality embryos (≥ Grade 4) the Oocyte Scoring System (TOS) (AUC±SE 0.863±0.044, oocyte score) provided significantly greater predictive value for clinical pregnancy compared to the embryo grade alone (AUC 0.646 ± 0.072, embryo grade) p = 0.015.

Finally, when ROC curves (Fig 3) were constructed to compare predictability of our oocyte-scoring system with our center’s traditional embryo-scoring system in predicting clinical pregnancy outcomes, the ROC curve for oocyte scoring trended favorably in comparison to embryo grade but did not reach significance (P = 0.12). However, when the analysis was restricted to the 69 transfers with good quality embryos (≥ Grade 4) the Oocyte Scoring System (TOS) (AUC±SE 0.863±0.044, oocyte score) provided significantly greater predictive value for clinical pregnancy compared to the embryo grade alone (AUC 0.646 ± 0.072, embryo grade) p = 0.015.

## Discussion

This study compared a newly developed oocyte scoring system with a traditional embryo scoring system. Relationships between egg quality (via oocyte- scoring) and resulting embryo quality (high vs. low, defined by ≥/< 8 cells on day-3 after fertilization embryo cell numbers and embryo grade) were evaluated. By comparing areas under the ROC curve, we, ultimately, were able to compare both systems’ ability to predict clinical pregnancy.

Our findings demonstrate that the TOS, incorporating cumulative effects of six oocyte parameters (morphology, size, ooplasm, ZP structure, PVS and morphology of the first PB), is directly predictive of embryo quality. It appears that better oocytes give rise to both improved day-3 cell numbers and embryo grade. For most parameters statistical significance was only seen among the top scores in univariate analysis. In other words, among oocytes with the best TOS, the odds of having ≥8-cell embryos, less fragmentation and a more favorable embryo grade significantly increase.

Detailed analyses of the six characterized oocyte parameters revealed that most of these oocyte characteristics were directly related to age. Only oocyte size proved to be a significant independent predictor of embryo quality after age adjustment. Since most of the parameters in the oocyte score varied directly with age, appearance of low scores in parameters aside from oocyte size among oocytes from young patients might have a different predictive value compared to those from older patients.

In the presence of good quality embryos patients with higher oocyte scores appeared more likely to achieve pregnancy after IVF. This suggests that the oocyte score might be helpful in identifying embryos most likely to succeed in pregnancy when all embryos appear to be of otherwise equal good quality.

This study, thus, indicates that within the purview of current embryology practice the potential predictive value of detailed oocyte assessments is underappreciated. Adoption of additional oocyte evaluation should be especially helpful in so-called good prognosis patient, where usually multiple good quality embryos are available for potential transfer [28].

The present findings should encourage replication by other IVF centers and, if confirmed, should lead to improved appreciation of oocyte scoring in routine embryology practice, establishing a simple new (and inexpensive) method of improving embryo selection. Since other proposed embryo selection technologies have so far largely failed to demonstrate effects on clinical pregnancy rates [28], a prospective confirmation of improved pregnancy outcomes with use of this oocyte scoring system would be important.

This analysis also raises significant further questions regarding current embryo selection protocols, largely based on embryo quality. This issue appears particularly relevant at a time when IVF laboratories are increasingly investing significant funds in automated embryo time-lapse incubation systems [11] in attempts to improve pregnancy chances.

Finally, this study uncovered several previously unreported parameters of human oocyte biology with predictive value in embryo selection. The range of variations, defined by TOS, have thus yielded possible leads for future studies of multiparametric analyses of human oocytes, incorporating basic morphological properties, currently routinely monitored in most IVF laboratories.

## Supporting Information

S1 Table Classification of oocyte quality by multiple parameters. (XLSX)

S2 Table Individual Patient and Cycle outcome characteristics. (XLSX)
